# Motivational Interviewing: A Guide for Medical Trainees

**DOI:** 10.1192/pb.bp.115.052647

**Published:** 2017-02

**Authors:** Ed Day

I first encountered motivational interviewing as a trainee when I read Miller and Rollnick's classic 1991 book *Motivational Interviewing: Preparing People to Change Addictive Behavior* and the key concepts have always resonated with me. Although it seems obvious that a man requiring major surgery due to cardiac disease should stop smoking, it is rarely helpful to insist that he does so. People have ambivalent feelings when it comes to changing entrenched behaviours and it is often better to elicit their own reasons for change. After all, it has been said that people believe what they hear themselves say. Perhaps because of its apparent simplicity, motivational interviewing has become an important technique for most UK addiction therapists and its influence has gradually spread to other areas of practice. Therefore, does the world need another book on motivational interviewing?

This book is written by a group of trainees spanning all specialties of medicine, with the goal of demonstrating how motivational interviewing can fundamentally improve the doctor-patient relationship. Motivational interviewing is a way of being rather than an intervention and the book reminded me of its roots in Carl Rogers' person-centred approach to therapy, based on building empathy, congruence and positive regard. As someone who bemoans the biomedical nature of British psychiatry, I was surprised that it succeeded in reawakening my interest in interviewing skills that not only elicit information but also provide therapeutic insights and direction.

Like the practice of psychiatry, motivational interviewing is straightforward to do but hard to do really well. It is not easy to learn from books and so the editors provide lots of dialogue to illustrate key points, and a series of videos on a linked website. They add personal reflections, as well as illustrations of the integration of motivational interviewing into electronic case records and its use in less familiar settings such as paediatrics. There is also a practical emphasis on how to teach and supervise motivational interviewing in the real world. Their enthusiasm for the subject was infectious and I was left in agreement that learning motivational interviewing should be a priority in medical education.

